# Protocol for a Scalable StoryListening Intervention for Grief-Related Loneliness During COVID-19

**DOI:** 10.1089/pmr.2023.0009

**Published:** 2023-08-08

**Authors:** Francesca Lynn Arnoldy, Matilda Garrido, Ann Wong, Susanna Pratt, Tess Braddish, Greg Brown, Maija Reblin, Donna Rizzo, Robert Gramling

**Affiliations:** ^1^ Professional and Clinical Education, University of Vermont, Burlington, Vermont, USA.; ^2^ University of Vermont Honor's College, Burlington, Vermont, USA.; ^3^ Office of Clinical Trials, University of Vermont, Burlington, Vermont, USA.; ^4^ Department of Family Medicine, University of Vermont, Burlington, Vermont, USA.; ^5^ Department of Civil and Environmental Engineering, University of Vermont, Burlington, Vermont, USA.

**Keywords:** doula, grief, listening, loneliness, storytelling

## Abstract

**Background:**

Social distancing during the COVID-19 pandemic limited how family, friends, and clinicians physically interacted with people who were dying and decreased communal opportunities for processing grief. These barriers can cause or exacerbate suffering due to loneliness while grieving.

**Purpose:**

In this article, we describe the protocol for a brief storytelling intervention designed to reduce loneliness among families, friends, and clinicians grieving the death of a person during the time of COVID-19.

**Methods:**

We trained four StoryListening doulas (SLDs) to hold a welcoming space and listen to stories with curiosity and openness. The intervention included a video StoryListening session and two brief questionnaires, filled out before and two weeks after the encounter, assessing loneliness and quality of life. During sessions, SLDs invited participants to share their story of loss in their own words and in as much detail as preferred. When participants felt a sense of story completion, SLDs shared validating statements and expressed gratitude to the participant for sharing. The video and audio for each participant's StoryListening encounter were recorded and the participant was offered an audio copy of their session.

## Introduction

### Grief-related loneliness in COVID-19 pandemic context

Death and loss during the COVID-19 pandemic occurred at a personal and societal level. Social distancing, travel restrictions, patient visitor limitations, fear of spreading or contracting COVID-19, and the use of personal protective equipment resulted in physically, emotionally, and spiritually separating the person who is dying from those caring about and for them. Subsequent communal opportunities for human connection that are typically available for those grieving, such as work, social events, funerals, wakes, and memorial services, became scarce. Cumulatively, the COVID-19 pandemic intensified bereaved families' and friends' feelings of deep disconnection,^1,2^ resulting in a particularly debilitating experience of “existential loneliness.”

Existential loneliness is the “immediate awareness of being fundamentally separated from other people and from the universe … and, especially when in crisis, not being met (communicated with) on a deep human (i.e., authentic) level.”^3(p.1322)^ Neimeyer and Lee observed that this type of loneliness amid COVID-19 independently predicted overall functional decline among people who experienced the death of a family or friend during the pandemic.^4^ Mortazavi et al. observed substantially higher burden of post-traumatic stress, complicated grief, and depression after one year of the COVID-19 pandemic compared with prepandemic rates.^5^

### Purpose of the storytelling intervention

The disruptions in interpersonal interactions during COVID-19 not only increased the incidence of existential loneliness, but they also diminished opportunities for grievers to verbally process their experiences of loss and grief. Storytelling is a near culturally ubiquitous way in which people find and share meaning about experiences in their lives. As described by Schenker et al., “Stories help us deal with surprises and upsets, make meaning out of chaos, clarify values, and build connections between past and future.”^6(p.452)^

Particular to the grieving process, telling one's grief story is essential to meaning reconstruction, which helps grievers reshape their views of the world and their place in it after a loss.^7^ Previous study by Barnato et al. observed that lay storytelling during the weeks to months after the person's death is both acceptable and comforting to those who are grieving.^8^

Telling one's story of loss to an interested listener can provide a vehicle for processing feelings of grief to make room for adjustment and loss reconciliation.^9^ End-of-life doulas are specifically trained to bear witness, without direction or judgment, to the stories and experiences of others, particularly during intense times of their lives.^10–12^ Rather than providing a therapeutic intervention with the goal of “fixing” or “curing,” doulas provide a safe welcoming space for the sharing of experiences, without prescribed prompting or facilitated analysis. Therefore, we designed a brief doula storytelling intervention to reduce the existential loneliness of grief among families, friends, and clinicians experiencing the death of a person during the time of COVID-19.

### Purpose of this report

The purpose of this report is to describe the protocol for a brief doula storytelling intervention that we found to be feasible and acceptable^13^ for reducing the existential loneliness of grief among families, friends, and clinicians who experienced the death of a person during the time of COVID-19.

### Potential applications in other contexts

The COVID-19 pandemic exacerbated an already growing worldwide burden of loneliness^14–16^ with profound implications for public health.^17–19^ As described in the forthcoming sections, this intervention is tailored specifically to the experience of grief and loss. However, we propose that the principles and structure of the StoryListening intervention offer promise for a scalable approach to loneliness reduction, regardless of the reasons for that loneliness.

Originally, we designed this as a face-to-face intervention. However, due to the COVID-19 social distancing restrictions, we modified the intervention to be delivered over televideo. As recently reported, participants found the intervention to be highly acceptable and impactful.^13^ Given the rapid growth,^20^ acceptability,^21,22^ and attention to quality^23^ of telehealth services, offering the StoryListening intervention over televideo may increase access for some isolated persons with transportation, in home caregiving or other barriers to a face-to-face encounter. At the same time, we suggest that a hybrid approach—telehealth and in-person options—may best serve our public health by honoring personal preferences^20^ and minimizing disparities in access to televideo healing services.^24–27^

## StoryListening Intervention

### Conceptual approach to listening

Doulas provide nonmedical emotional support to those facing times of intensity, such as birth, death, and grief. Doulas cultivate a nonanxious presence, validate through attentive listening, hold trust in the inherent wisdom and strength of others, adopt a stance of engaged neutrality and acceptance, allow and welcome silence, respect perspectives and beliefs, and normalize experiences by acknowledging universal suffering as well as commonalities in loss.

A StoryListening doula (SLD) focuses specifically on inviting and listening to experiences of the bereaved, as perceived and narrated by each storyteller. SLDs are nondirective and let the participant guide the depth and direction of each session. SLDs respect grief as a natural, ongoing process that is unique to each person for each loss. Doulas do not advise, proselytize, or provide medical advice or explanations, psychotherapy (longitudinal support or directional guidance in the forms of cognitive restructuring, reframing, or reconceptualizing), solutions, arguments, agreements, or false reassurances.

Unlike narrative psychotherapy or critical incident debrief, SLDs do not direct the conversation or assist the storyteller with analysis and reconciliation of the traumatic loss or event or their experiences of it.^28^ No future coping strategies are prescribed, nor are reactions or feelings classified based on traditional psychological models. The intervention provides a container for candid reflection as directed and managed by the storyteller themselves, with acknowledgment, recognition, and careful prompting, if appropriate, from the SLD.

### Preparing the encounter setting

SLDs prepared their space and themselves for each StoryListening session. In terms of the recording setting, it needed to be quiet, uncluttered, and free from personal photographs as well as religious or spiritual items (e.g., jewelry). SLDs wore professional attire and had the research project brochure nearby in case there were questions. Each doula developed his or her own personal practice of slowing down and centering with intention before beginning the sessions. These practices might have included breath work, a mantra, and mindfully setting aside personal concerns or stressors.

### Initiating the encounter

All StoryListening encounters happened over televideo. Once participants entered the videoconference “waiting room,” the study coordinator recorded the date and participant number, and then made the SLD the video conference “host.” Occasionally, participants needed assistance with technology (camera, microphone). Once settled, the SLDs initiated introductions and kept small talk to a minimum while relaying a brief overview of what to expect, the doula role, and this check-in: “If someone is feeling really overwhelmed in their grief, then talking about it can sometimes be distressing. Taking into consideration how you're feeling today, does now still seem like a good time for our session?”

If the participants confirmed their readiness, the SLD opened space for storytelling by prompting either, “In your own words and in as much detail as you'd like, can you share with me your experience caring for patients during the pandemic?” (for clinicians and staff), or, “In your own words and in as much detail as you'd like, can you share with me the story of your recent loss?” (for friends or family members).

### Process of listening

Many stories unfolded without additional prompting. SLDs relied often on silence to provide ample opportunities for processing and expression. If/when responses seemed beneficial, SLDs utilized invitations to expand (“Would you like to tell me/say more about…”), and/or invitations to clarify (“That sounds…,” “It sounds like…,” “I hear that you…,” or “I hear you saying…”). To maintain an other-oriented lens, SLDs avoided phrases such as “I know” and “I understand.”

If/when doula prompts seemed beneficial or were requested, SLDs modified versions of the following examples: *What was most difficult? What was most surprising? What were the most meaningful or important moments for you? What memories do you hold most heavily? What memories do you hold most dear?* When assessing a participant's sense of completion, an SLD would ask: *Was there anything else you were hoping to share today?*

### Recognition and response to distress

Many participants emoted and expressed distress to varying degrees through tears, sighs, silence, expressions of exasperation, etc. These are expected reactions to loss and stress. If a participant, instead, displayed signs of more extreme signs of acute distress, doulas were trained to (1) gently pause/interrupt their story, verbalize concern for the participant's well-being, and suggest not completing the session, (2) ask the participants to remain in the televideo platform and explain they were calling for support, and (3) page the on-call study physician to join the televideo session.

### Closing

To maintain commitment to a nondirective approach, doulas avoided creating a disingenuously positive summary when sessions were closing. SLDs identified key themes (if/when participant has not already done so), shared validation statements by rewording some of the major sentiments or insights voiced by the storyteller (sources of pride and/or meaning and any coping mechanisms recognized), expressed gratitude to the participant for sharing their story, and relayed a version of this sentiment: *Sometimes, after talking about loss and grief, people find themselves continuing to think about the experience in the days to come. Some people find it useful to have a journal (or just blank paper) nearby so they can write down what is on their mind. I encourage you to take really good care of yourself in ways that feel nourishing*.

Finally, the doula informed the participants they would be hearing from the study coordinator and would receive an audio version of the recording in the weeks to come.

### Sharing recording

Each StoryListening encounter was recorded in both a video file and an audio file. Each participant was offered an audio copy of his or her session. For those who wished to receive a copy, the digital file was shared with the participant through a secure online file transfer service.

## Interventionist Training, Support, and Fidelity Assurance

### Training

As a prerequisite for the interventionist role, all four SLDs first successfully completed an eight-week online professional certificate course at a prominent End-of-Life Doula training program that is available internationally (learn.uvm.edu/program/end-of-life-doula-at-uvm).

The interventionist doulas then participated in an initial group training session to learn about the purpose and organization of this study. The interventionist team reviewed the institutional review board (IRB)-approved research protocols, technology requirements (earbuds and computer/camera), StoryListening session components (timing, role of study coordinator, and back-up assistance), scene/setting guidelines for SLDs, and next steps (completing IRB training modules, sending calendar availability for sessions, and scheduling future team meetings).

The second group training session began with reviewing “The Ins of Holding and Creating Space”^11^ and proceeded to focus on conducting sessions (self-preparation, shifting in, how to open and close sessions, suggested questions, and shifting out postsession), special situations (potential participant requests, reasons to cancel/postpone/stop a session, acute crises, and mandated reporting), and doula approaches (phrases to utilize/avoid and outline of role and scope). SLDs also prepared by holding practice sessions with one another with each doula having a chance to both share and listen to grief stories.

Subsequent group training sessions addressed specific topics such as the importance of the grief check-in at the start of sessions, appropriate boundaries, and how to identify and manage hidden assumptions/agendas, conditioning, triggers, and shadow sides. Other topics included appropriate doula responses if participants were to seek direct guidance, voice intense admissions (guilt, shame, neglect, anger, and resentment), and express perceptions of mistreatment or inadequate care related to the death or disclose trauma. To prevent fatigue and/or emotional harm to interventionist, the final group training session focused on defining and recognizing secondary traumatic stress as well as formulating individual and team strategies to bolster resilience.

In addition to the group training series, the intervention director scheduled individual meetings after each interventionist's first StoryListening session to review the recording and discuss general concerns, reflections, questions, and goals.

### Standard operating procedures reference

We created a standard operating procedures (SOPs) manual, which outlines and comprehensively describes the Doula Training Sessions (group and individual), Research Procedures, Session Prompts for Doulas, the StoryListening Doula Recruitment Form, and the StoryListening Enrollment Visit Reminder Checklist. The SOP manual functioned as a point of immediate “field” reference for SLDs, study coordinators and investigators, and as a living document updated as new situations arose throughout the study to provide a basis for subsequent implementation by other teams. (Please contact the corresponding author of this article for information about accessing the SOP for research implementation.)

### Debrief

To sustain the well-being and effectiveness of the SLDs, the interventionist team met regularly (weekly or biweekly) to debrief and process. These meetings provided a time for honest disclosures about personal responses to sessions, questions about the project, clarification on the doula role, an exploration of patterns, and the sharing of unexpected occurrences. In the case of a particular theme, with the SLD's permission, the group watched a particular recording (or segment of a recording) separately and debriefed the session together, offering supportive insights.

### Fidelity maintenance

To ensure fidelity, study leads regularly reviewed recordings of StoryListening sessions, both randomly selected and any identified by an SLD as being challenging or presenting a novel situation. Based on the nature of the feedback required to maintain intervention fidelity, the intervention director met with individual SLDs and/or the interventionist team as a group to discuss issues that arose and role-play new or challenging scenarios, and also updated the SOP manual as needed.

## Study Population

### Eligibility

Adult English-speaking family members, friends, or clinicians of any person who died of any cause during the COVID-19 pandemic were eligible participants for this study. Participants could reside anywhere in the United States. For family member and friend participants, eligibility did not require physical or virtual presence near the time of death. For clinician participants, eligibility required only that they were professionally involved in the person's care preceding their death. Participation required access to a telecommunication device (e.g., phone, tablet, or computer) and Internet connectivity sufficient to support televideo. All participants received the same StoryListening intervention.

### Recruitment

We used multiple methods for recruiting participants. First, we posted the study flyer in public facing venues, including grocery stores, religious organizations, universities, hospitals, and community bulletin boards near the primary study site. Second, we shared electronic versions of the same flyer on local community news websites, national professional clinical organizations (e.g., American Academy of Hospice and Palliative Medicine), and with national clinical leaders in end-of life research. Third, we provided study flyers and brochures to leaders of hospitals (e.g., chief medical and nursing officers), clinical departments (e.g., intensive care units and emergency departments), hospice agencies, and long-term care facilities in our local health care network.

### Informed consent

Potential participants expressed interest by directly contacting the StoryListening study coordinator through e-mail or telephone. The study coordinator shared electronic informed consent documents with potential participants and reviewed them together by telephone before obtaining informed verbal consent. None of the study recruitment or data collection procedures involved accessing health care records for the study participants or for the person who died.

## Data Collection

### Participant self-report

The project study coordinators administered two brief telephone questionnaires with study participants. The pre-encounter questionnaire (18 items) occurred at the time of study enrollment when participants scheduled their StoryListening encounter (usually within two weeks of enrollment). The postencounter questionnaire (15 items) occurred approximately two weeks after the StoryListening encounter. The time to complete each questionnaire was typically 5–10 minutes.

### Video and audio recording

The video and audio for each participant's StoryListening encounter were recorded using the endemic function in televideo software.

## Measures

### Loneliness and quality of life

Participants completed the same brief measures of loneliness and quality of life at the time of enrollment and at the two-week follow-up visit. We assessed loneliness using the three-item short form of the University of California, Los Angeles (UCLA) Loneliness Survey.^29^ We used six items from the McGill Quality of Life Questionnaire representing the subscales for global quality of life (QOL) (one item), existential QOL (two items), and social QOL (three items).^30^

### Open-ended questions

At the two-week follow-up telephone interview, participants were asked to respond to the three brief open-ended questions to explore acceptability of the intervention. The first focused on the experience of the storytelling encounter (*In your own words, what was it like to share your experience during the* StoryListening *visit?*), the second on perceived effect of telling one's story (*Did you find the* StoryListening *visit had any impact on your quality of life? If so, in what ways?*), and the third on the use of their story audio file (*Did you use the recording of the* StoryListening *visit? If so, how was that experience?*). The study interviewer recorded participant responses in writing, as close to verbatim as possible.

### Participant characteristics

Participants self-reported their age, gender identity, race, ethnicity, educational attainment, religious affiliation, and financial strain. Family and friend participants reported their relationship with the person who died, when they died, and the setting in which they died. Clinician participants reported their clinical discipline, the setting in which they care for people who are near death, and approximate number of patients for whom they cared in the hours to days before dying during COVID-19.

Description of the participant samples and their experience of the intervention are available elsewhere.^13^

## Human Subjects

Study procedures were approved by the institutional review board at the University of Vermont (Protocol ID: 00000925).

## Funding Information

This study was funded by the Holly and Bob Miller Endowed Chair in Palliative Medicine and the Kate Laud Story Fund at the University of Vermont.

## Abbreviations Used

SLDs: StoryListening doulas
SOPs: standard operating procedures
